# Efficacy and safety of tenofovir in preventing mother-to-infant transmission of hepatitis B virus: a meta-analysis based on 6 studies from China and 3 studies from other countries

**DOI:** 10.1186/s12876-018-0847-2

**Published:** 2018-08-02

**Authors:** Wenhui Li, Li Jia, Xin Zhao, Xiaoyuan Wu, Hongxia Tang

**Affiliations:** 1 Department of Infectious Disease, Children’s Hospital of Hebei Province, 133 South Jianhua Street, Shijiazhuang, 050031 Hebei China; 2 Department of Neurology, Children’s Hospital of Hebei Province, 133 South Jianhua Street, Shijiazhuang, 050031 Hebei China

**Keywords:** Hepatitis B, Tenofovir, Vertical transmission, Meta-analysis

## Abstract

**Background:**

The vertical transmission of HBV from mothers to their infants at birth or in early infancy has a significant role in the endemicity of HBV infection. Tenofovir is one of the most potent anti-HBV agents with a high genetic barrier to resistance. The study is to evaluate the efficacy of tenofovir in preventing perinatal HBV transmission, as well as monitoring safety for mothers and infants.

**Methods:**

PubMed, Embase, Web of Science, and CNKI (National Knowledge Infrastructure, China) database were systematically reviewed for studies that compared the efficacy and safety of tenofovir with other treatments. Pooled estimates were expressed with weight mean difference (WMD) with 95% confidence intervals (95% CIs) and risk ratio (RR) with 95% CIs.

**Results:**

Nine studies involving 1046 pregnant patients met the inclusion criteria and were included in this meta-analysis. Compared with other treatments, tenofovir significantly reduced maternal HBV DNA levels (WMD = 2.33 log_10_ IU/mL, 95% CI: 1.01, 3.64; *P* < 0.001), infant HBsAg positivity rate (RR = 0.25, 95% CI: 0.16, 0.38; *P* < 0.001), infant HBeAg positivity rate (RR = 0.26, 95% CI: 0.14, 0.48; *P* < 0.001), infant HBV DNA positivity rate (RR = 0.15, 95% CI: 0.07, 0.31; *P* < 0.001), and immunoprophylaxis failure rate (RR = 0.31, 95% CI: 0.13, 0.73; *P* = 0.008). Moreover, maternal and infant safety profiles, including ALT, CK, and Cr were comparable between tenofovir and other treatment groups.

**Conclusion:**

Based on the current evidence, our study suggested that tenofovir significantly reduced the rate of vertical transmission of HBV, as well as the HBV DNA levels in HBV-infected mothers. Moreover, tenofovir was safe and tolerable for both mothers and their infants.

## Background

Chronic hepatitis B virus (HBV) infection is a serious threat to public health and is associated with cirrhosis and liver cancer [1, 2]. Although the universal infant immunization reduces 80–90% of chronic HBV infection, the active/passive immunoprophylaxis has not eradicated mother-to-infant HBV transmission [3–5]. The vertical transmission of HBV from mothers to their infants at birth or in early infancy has a significant role in the endemicity of HBV infection [6]. Moreover, after immunoprophylaxis, children with HBV infection have a higher risk of developing hepatocellular carcinoma [7, 8].

HBV immunoprophylaxis failure is influenced by maternal hepatitis B e antigen (HBeAg) positivity and viral load [9, 10]. Several studies have demonstrated a positive correlation between high maternal serum HBV DNA levels and an increased risk for vaccination breakthrough [11, 12]. Thus, these data have introduced the idea of antiviral therapy in pregnant women with high HBV DNA levels to help achieve the goal of global eradication of HBV infection.

Three oral anti-HBV agents have been approved by United States Food and Drug Administration for the preventing of mother-to-infant transmission. These agents included lamivudine, telbivudine, and tenofovir. Lamivudine has been reported to reduce 72–78% risk of vertical transmission compared with passive/active immunization alone [13, 14]. However, it could lead to the emergence of lamivudine-resistant mutants because of its lower genetic barrier to resistance [15]. Telbivudine has been proven to prevent immunoprophylaxis failure in all cases treated; however, the increased risk of resistance limited its prolonged therapy [6, 16].

Tenofovir is one of the most potent anti-HBV agents with a high genetic barrier to resistance [17]. A small but growing body of evidence has suggested that tenofovir may reduce the risk of mother-to-child transmission, however, their results remain inconsistent [6, 13, 16, 18–20]. To increase power and precision, we conducted this meta-analysis based on relevant studies to assess the efficacy of tenofovir in reducing perinatal HBV transmission, as well as monitoring safety for mothers and infants.

## Methods

### Literature search

We conducted this meta-analysis in accordance with the Preferred Reporting Items for Systematic Reviews and meta-analysis (PRISMA) criteria [21]. PubMed, Embase, Web of Science, and CNKI (National Knowledge Infrastructure, China) database from inception to January 16, 2018 were searched to identify relevant studies. The search was limited to human subject, and no language restriction was imposed. Search terms were as follows: (“hepatitis b virus” [MeSH Terms] OR “hepatitis b virus” [All Fields]) AND (“infectious disease transmission, vertical”[MeSH Terms] OR (“infectious” [All Fields] AND “disease” [All Fields] AND “transmission” [All Fields] AND “vertical” [All Fields]) OR “vertical infectious disease transmission”[All Fields] OR (“mother” [All Fields] AND “child” [All Fields] AND “transmission” [All Fields]) OR “mother to child transmission” [All Fields]) AND (“telbivudine” [Supplementary Concept] OR “telbivudine” [All Fields]). In addition, we also searched the lists of included studies and reviews to identify other potentially eligible studies.

### Review strategy

We used Endnote (version X, Thomson Reuters, Inc., Philadelphia, PA) bibliographic software to create an electronic library of citations identified in the literature search. PubMed, Embase, and Web of Science searches were performed using Endnote, and duplicate records were deleted. Two independent investigators were trained to conduct the abstract review and full-text review thereafter. Disagreements between them were resolved by discussion and consensus. The inter-reviewer agreements were calculated using the Cohen K statistic [22].

### Study inclusion and exclusion criteria

Studies were included in this meta-analysis if they met the following inclusion criteria: (1) study design: randomized controlled trial (RCT), or case-control study, or cohort study; (2) study population: pregnant women 20 to 35 years of age who had chronic HBV infection, were HBeAg-positive; (3) comparison intervention: tenofovir and other treatments; (4) outcome measure: mother to child transmission rate, maternal HBV DNA levels, maternal and infant safety outcomes. If several publications were from the same clinical trial, we only included the most informative article or the longest follow-up study to avoid duplication of information.

Studies were excluded in this meta-analysis if they were published with the study type of editorials, comments, reviews, letters or unrelated with our topics.

### Data extraction and quality assessment

We used a standardized data-extraction sheet to extract the following information: first author, year of publication, number of patients in each arm, age of the pregnant women, population characteristics, study design, and the outcome of interest.

We assessed the risk of bias in RCT using the method recommended by Cochrane Collaboration [23]. This method consisted five items to evaluate the quality of study, including blinding, method of randomization, allocation concealment, follow-up, and intention-to-treat analysis [23]. Each study was considered to be high, low, or unclear risk of bias according to the above-mentioned criteria.

The quality of non-randomized studies was assessed using a modified Newcastle-Ottawa scale (NOS) [24]. This scale consisted three items that descried patient selection, comparability of the tenofovir and other treatment groups, and outcome assessment. The total scale ranged from 0 to 9 points. Study with a scale of more than 5 points was considered as high quality [24].

### Statistical analysis

We calculated weight mean difference (WMD) with 95% confidence interval (95% CI) for continuous outcomes, and risk ratio (RR) with 95% CI for dichotomous outcomes. Before the data were pooled, we first tested the heterogeneity between the included studies, using I^2^ statistic and Cochrane Q chi-square test [25]. A value of I^2^ more than 50%, or the value of P less than 0.10 indicated significant heterogeneity across the included studies [25]. When significant heterogeneity was identified, a random-effects model (DerSimonian-Laird method) [26] was used to summary the estimates; otherwise, a fixed-effects model (Mantel-Haenszel method) [27] was applied. When considerable heterogeneity was found, sensitivity analysis was performed by omitting one study in each turn to explore the influence of a single study on the overall estimate. Publication bias was evaluated by Begg [28] and Egger’s [29] test. A *P* value less than 0.05 was judged as statistically significant, except where otherwise specified. All statistical analyses were performed using STATA, version 12.0 (Stata Corporation, College Station, TX, USA).

## Results

### Identification of eligible studies

**Fig. 1:**
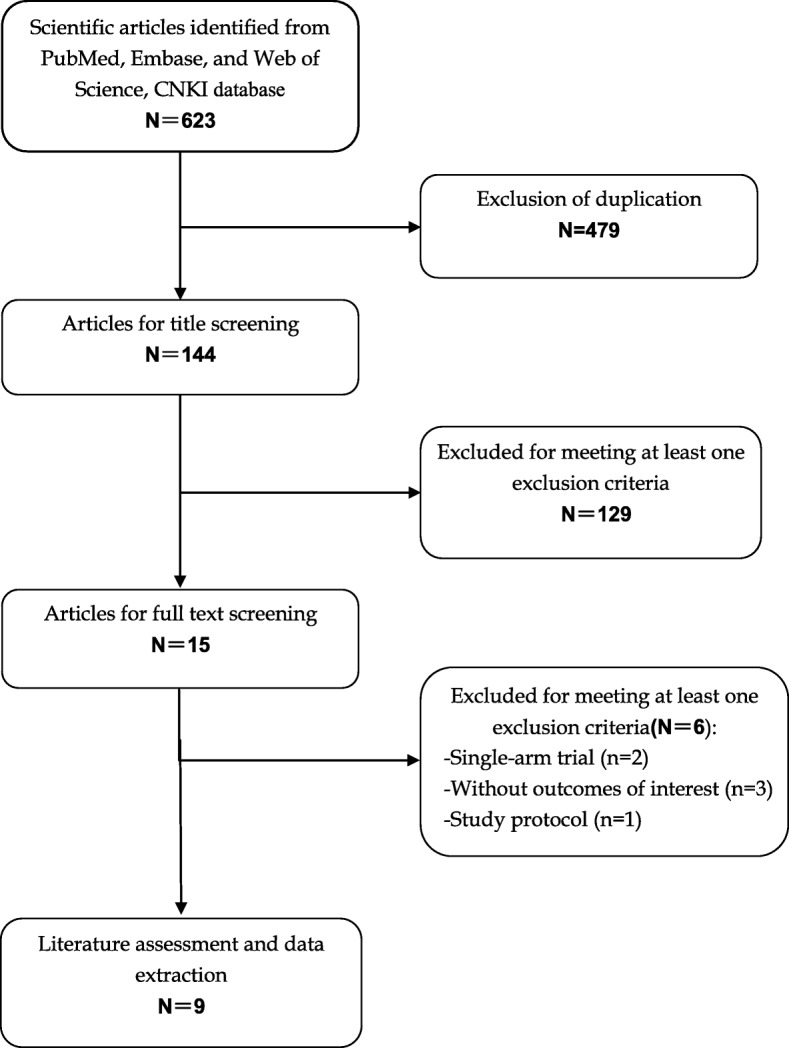
Eligibility of studies for inclusion in meta-analysis

A total of 623 relevant records were identified from the database search. Of these, 479 studies were excluded because of duplicate records, and 129 studies were excluded after a review of title/abstract. Then remaining 15 studies were scrutinized for full-text information review, however, 6 of them were excluded because 2 were single-arm study design [30, 31], 3 did not provide outcomes of our interest [32–34], and one was a study protocol [35]. Finally, nine studies [20, 36–43] that met the inclusion criteria were included in this meta-analysis (Fig. 1). The Cohen statistic K for agreement on study inclusion was 0.91.

### Characteristics of eligible studies

The main characteristics of included studies were presented in Table 1. These studies were published between 2013 and 2017. The sample size ranged from 40 to 153. Among these studies, six were conducted in China [36, 37, 40–43], each one in Austria [20], Turkey [38] and Canada [39], respectively. Of the included studies, eight were cohort studies [20, 36, 38–43], and one was RCT [37]. All the pregnant women had HBV DNA ≥6.0 log_10_ IU/mL. The tenofovir was administrated with a dosage of 300 daily. The timing of pregnancy when tenofovir was started varied greatly across the included studies, with 18–32 weeks of gestation in seven studies [20, 36–41], and 6 or 8 weeks in two studies [42, 43]. All the studies [20, 36–43] compared tenofovir with no medication or usual medical care without antiviral therapy, and two studies compared it with lamivudine [20] and telbivudine [40] additionally. Since these two studies provided two sets of data: tenofovir versus no therapy, tenofovir versus lamivudine/ telbivudine, we extracted all these data for data analysis.

**Table 1:** Baseline characteristics of patients in the trials included in the meta-analysis

Study	Country	Study design	Treatment regimen	No. of patients	Pregnancy time when tenofovir was started (w)	Age (mean ± SD, y)	HBV DNA (log10 IU/ml)	NOS score
Greenup AJ [20]	Austria	Cohort	Tenofovir 300 mg daily	58	30–32	30 ± 8.5	7.94 ± 0.78	7
			Lamivudine 100 mg daily	52		28 ± 5.3	7.72 ± 0.61	
			No medication	20		28 ± 5	8 ± 0.04	
Chen HL [36]	China	Cohort	Tenofovir 300 mg daily	62	32	32.41 ± 3.12	8.25 ± 0.45	9
			No medication	56		32.45 ± 3.2	8.24 ± 0.35	
Pan CQ [37]	China	RCT	Tenofovir 300 mg daily	97	30–32	27.4 ± 3.0	8.2 ± 0.5	NA
			Usual care	100		26.8 ± 3.0	8.0 ± 0.7	
Celen MK [38]	Turkey	Cohort	Tenofovir 300 mg daily	21	18–27	28.2 ± 4.1	8.28	6
			Untreated	24		26.9 ± 2.9	8.31	
Kochaksaraei GS [39]	Canada	Cohort	Tenofovir 300 mg daily	23	28 (21–32)	30(28–34)	7.7(3.2–8.1)	6
			Untreated	138		32(29–36)	2.3(1.6–3.1)	
Chen WJ [40]	China	Cohort	Tenofovir 300 mg daily	30	23.9 ± 1.9	28.67 ± 5.71	≥6.0	7
			Telbivudine 600 mg daily	79		31.13 ± 6.27	≥6.0	
			Untreated	44		29.86 ± 5.05	≥6.0	
Wan JY [41]	China	Cohort	Tenofovir 300 mg daily	74	28	28.5 ± 4.2	7.69 ± 0.54	8
			Untreated	42		27.9 ± 4.0	7.57 ± 0.48	
Qi LW [42]	China	Cohort	Tenofovir 300 mg daily	54	6	27.5 ± 3.6	7.01 ± 0.98	7
			Untreated	32		26.4 ± 3.1	6.51 ± 1.03	
Deng J [43]	China	Cohort	Tenofovir 300 mg daily	20	10	26.15 ± 2.56	NR	7
			Untreated	20		28.32 ± 2.45	NR	

*Abbreviation*: *SD* standard deviation, *NR* not reported, *NA* not available, *NOS* Newcastle-Ottawa scale

### Quality of included studies

The quality of cohort studies was assessed by the modified NOS. The scores of these studies ranged from 6 to 9, indicating that these cohort studies were of high quality (Table 1). For the only one RCT [37], since the blinding of participants and personnel and blinding of outcome assessors were not reported, it was classified as being at unclear risk of bias.

### Maternal effects

#### Maternal HBV DNA suppression

**Fig. 2:**
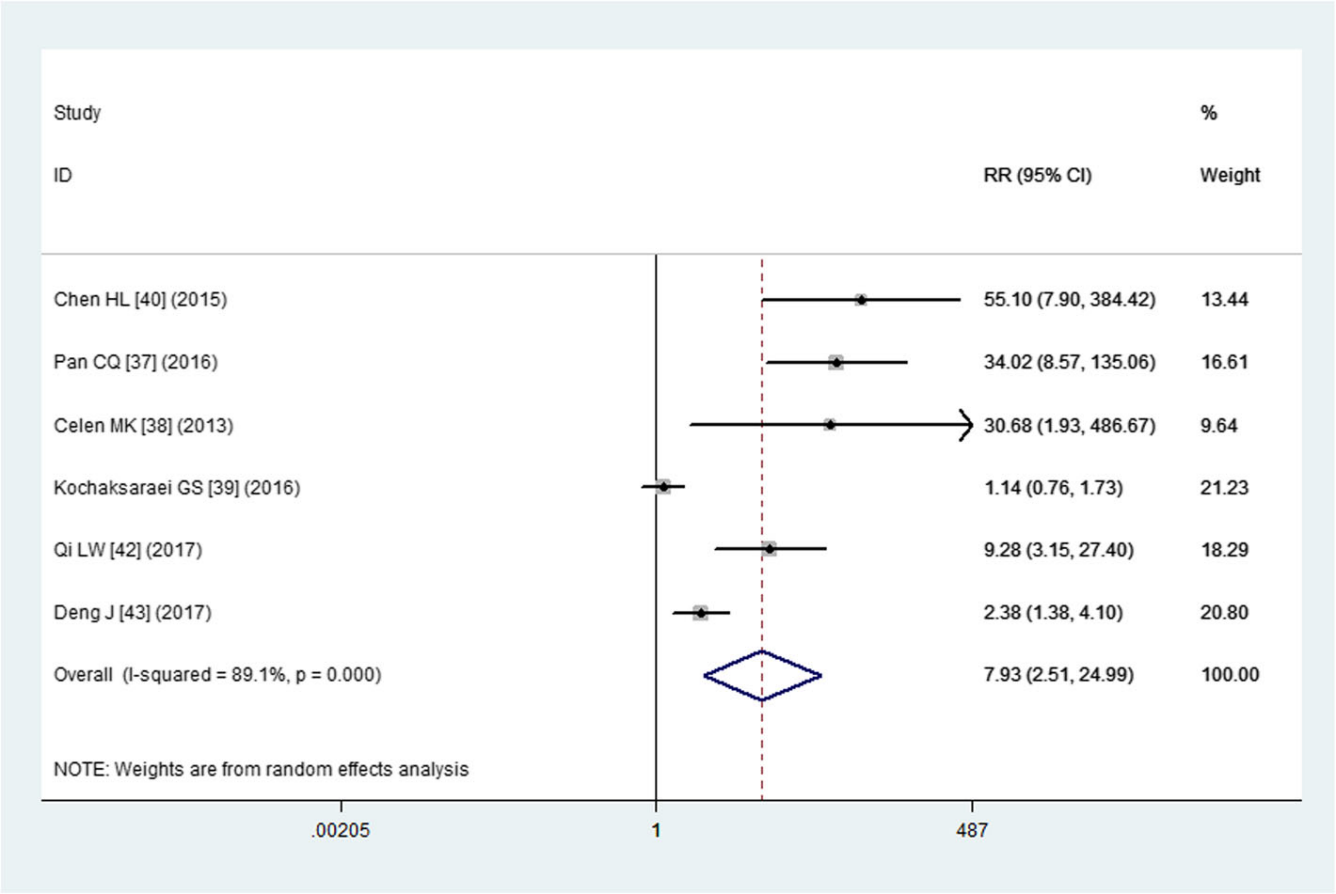
Forest plot showing the effect of tenofovir on the maternal HBV DNA suppression

Six studies reported the data of maternal HBV DNA suppression [36–39, 42, 43]. The pooled estimate demonstrated that tenofovir was associated with an improvement in the maternal HBV DNA suppression rate (RR = 7.93, 95% CI: 2.51, 24.99; *P* < 0.001) (Fig. 2). The test for heterogeneity was significant (*P* < 0.001, I^2^ = 89.1%). Therefore, we conducted sensitivity analysis to explore the potential sources of heterogeneity. When we excluded the study with outlier [39], the pooled estimate changed a little (RR = 13.70, 95% CI: 3.45, 54.30; *P* < 0.001), but the heterogeneity was still observed (*P* < 0.001, I^2^ = 82.9%). When we excluded the two trials [38, 43] that had a relatively small sample size (*N* < 50), the overall estimates did not change substantially (RR = 10.61, 95% CI: 1.46, 76.87; *P* = 0.019), but the heterogeneity was still present (*P* < 0.001, I^2^ = 92.9%). We also further excluded any single study; however, this did not materially alter the overall estimate (data not shown).

#### Maternal HBV DNA level reduction

**Fig. 3:**
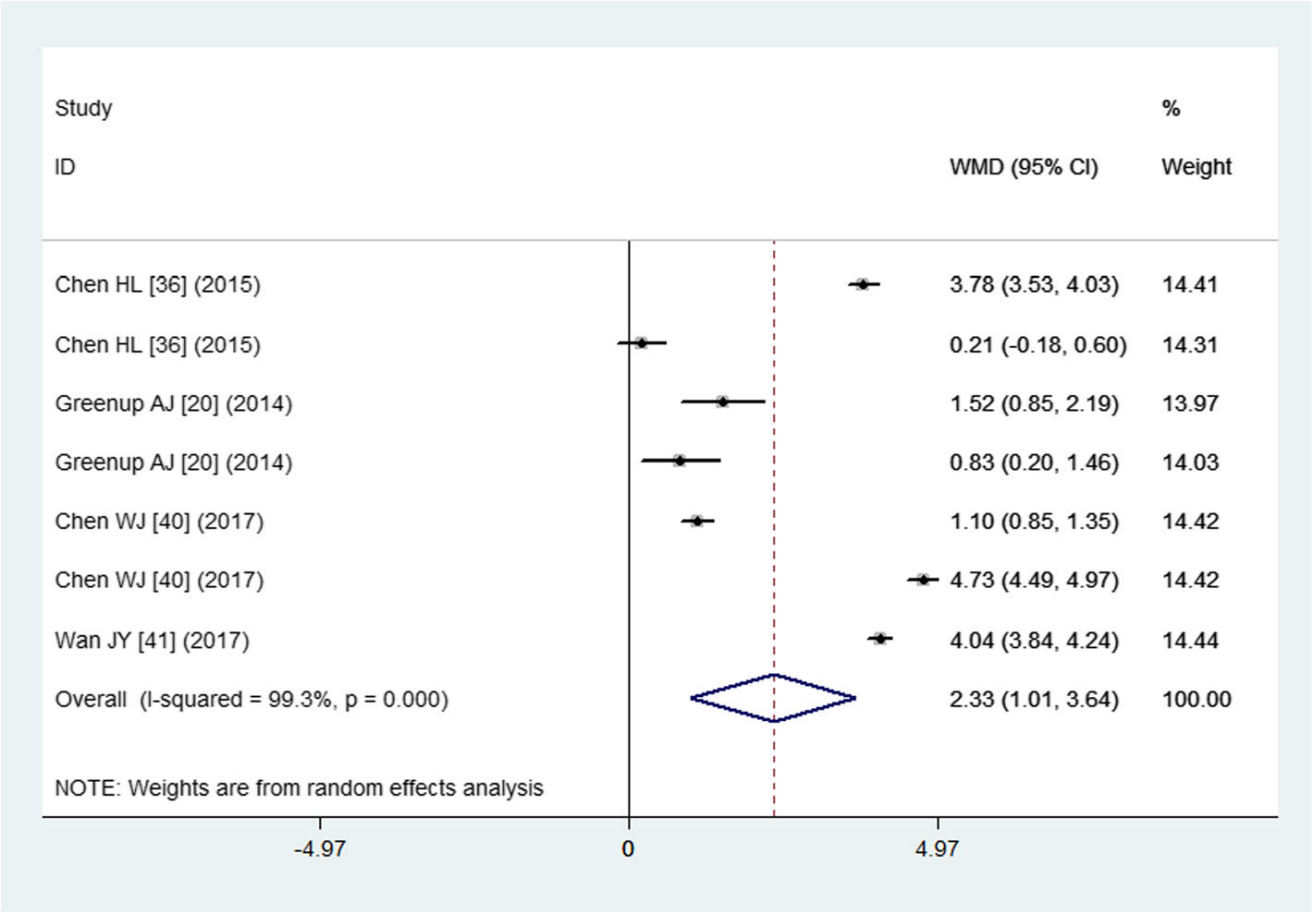
Forest plot showing the effect of tenofovir on the maternal HBV DNA level reduction

Four studies reported the data of maternal HBV DNA level reduction [20, 36, 40, 41]. The aggregated result suggested that, tenofovir was associated with a significant reduction in maternal HBV DNA level (WMD = 2.33 log_10_ IU/mL, 95% CI: 1.01, 3.64; *P* < 0.001) (Fig. 3). There was significant heterogeneity among the included studies (*P* < 0.001, I^2^ = 99.1%). Therefore, we conducted sensitivity. When we excluded the study with outlier [36], the pooled estimate did not change substantially (WMD = 2.69 log_10_ IU/mL, 95% CI: 1.41, 3.96; *P* < 0.001), however, the heterogeneity was still present (*P* < 0.001, I^2^ = 99.1%). When we excluded the study with small sample size (*N* = 74) [[40]], the pooled result did not alter substantially (WMD = 2.59 log_10_ IU/mL, 95% CI: 1.45, 3.73; *P* < 0.001), but the evidence of heterogeneity was still observed among the remaining studies (*P* < 0.001, I^2^ = 97.7%).

#### Maternal HBeAg seroconversion

Two studies reported the data of maternal HBeAg seroconversion [36, 37]. The pooled estimate showed that tenofovir had a similar maternal HBeAg seroconversion rate with other treatments (RR = 2.87, 95% CI: 0.45, 18.49; *P* = 0.266). The test for heterogeneity was not significant (*P* = 0.369, I^2^ = 0.0%).

#### Maternal ALT, CK and Cr elevation

**Fig. 4:**
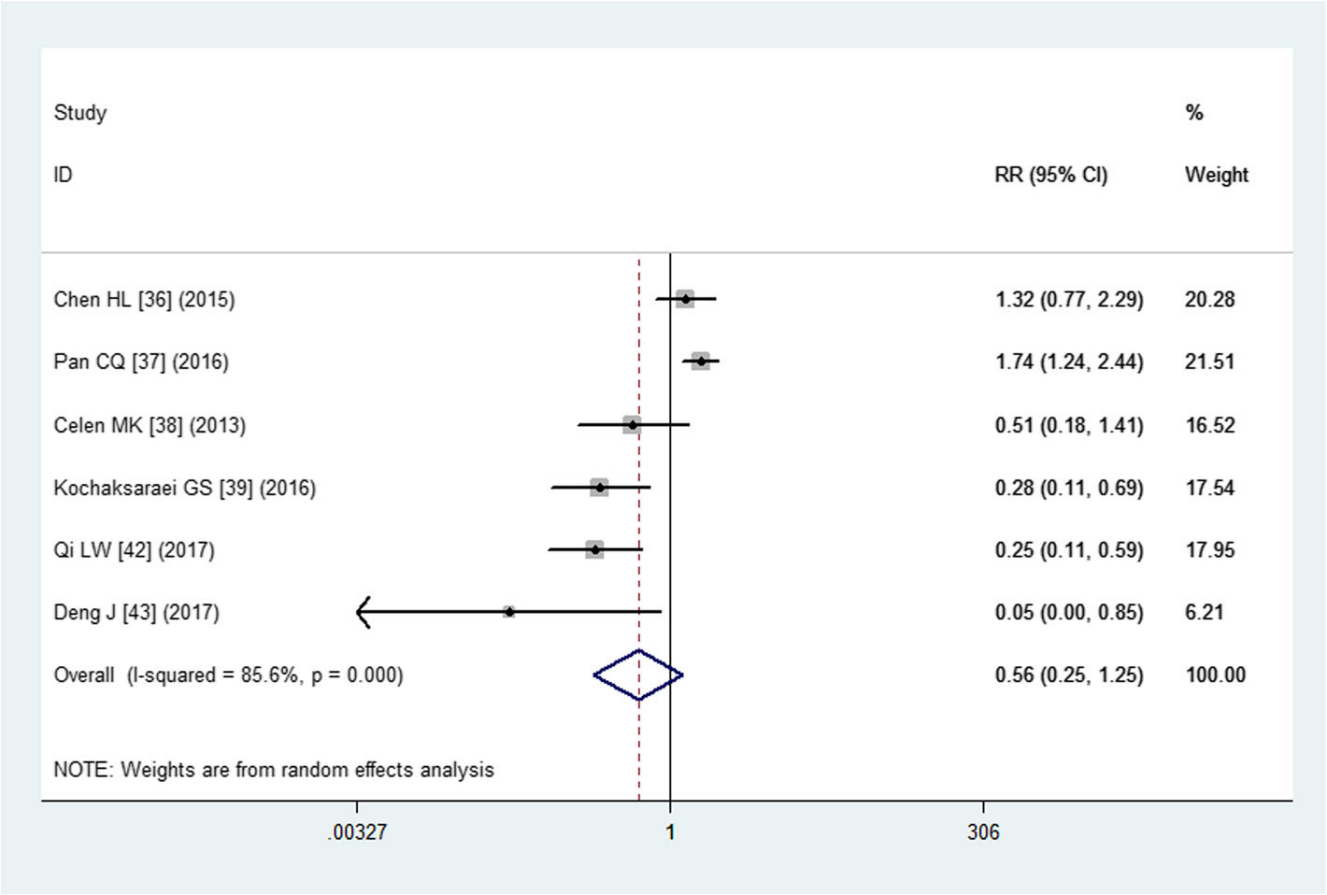
Forest plot showing the effect of tenofovir on the maternal ALT elevation

Six studies reported the data of ALT, CK, and Cr elevation [36–39, 42, 43]. The pooled estimate demonstrated that patients treated with tenofovir had a comparable ALT elevation than those with other treatments (RR = 0.56, 95% CI: 0.25, 1.25; *P* = 0.157) (Fig. 4). Moreover, the ALT levels in the two groups were not significant difference (WMD = 9.37 U/L, 95% CI: -13.50, 32.24; *P* = 0.130).

The CK and Cr levels in the two groups were also comparable. Patients treated with tenofovir developed a similar CK (WMD = 4.45 mg/dL, 95% CI: -1.51, 10.41; *P* = 0.143) and Cr (WMD = − 0.08 mg/dL, 95% CI: -0.23, 0.07; *P* = 0.316) levels as those treated with other treatment.

### Infant effects

#### Infant HBsAg positivity

**Fig. 5:**
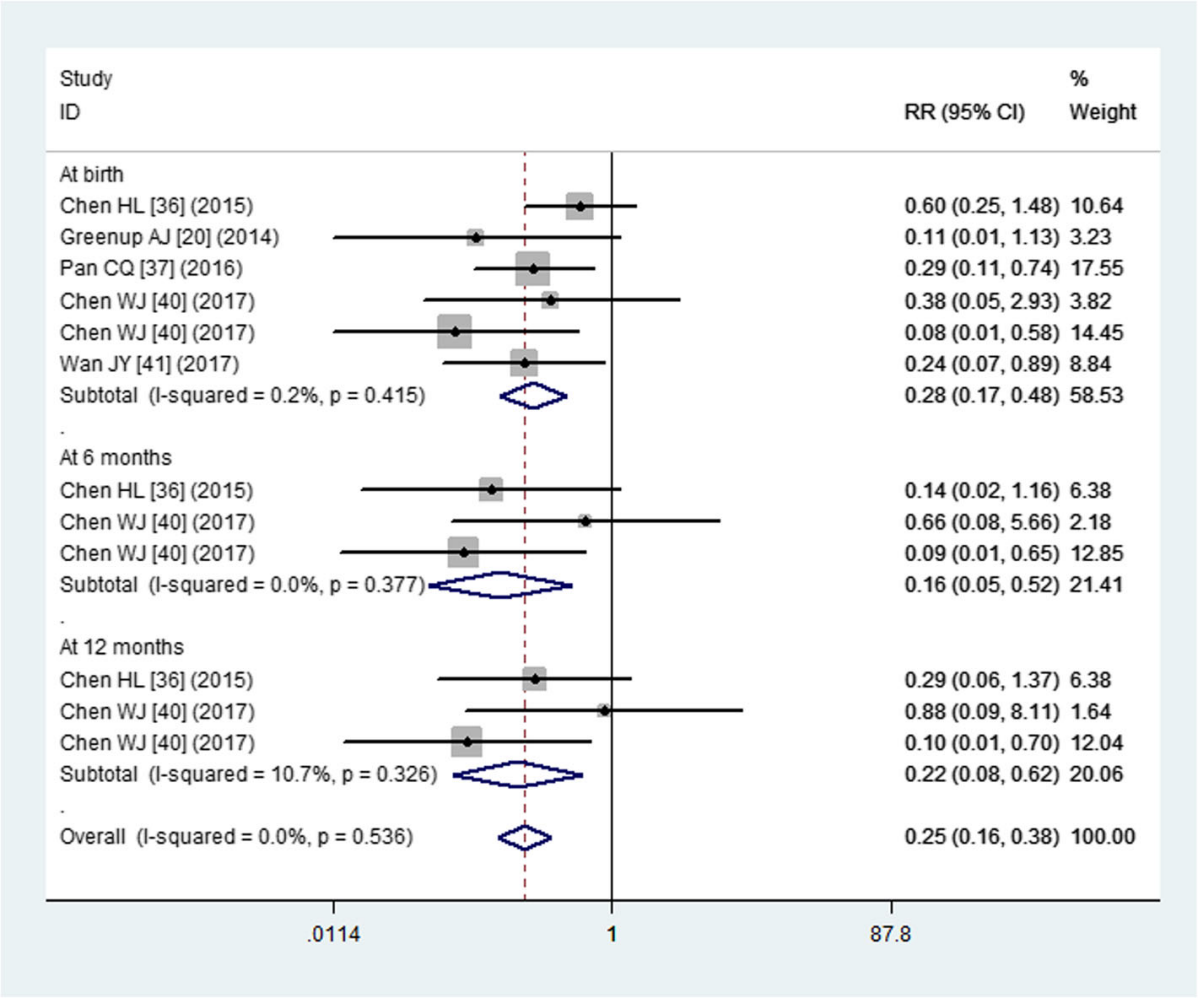
Forest plot showing the effect of tenofovir on the infant HBsAg positivity

Five studies reported the data of infant HBsAg positivity [20, 36, 37, 40, 41]. The pooled result suggested that, infants in the tenofovir group had a significant lower rate of HBsAg positivity compared with the control group (RR = 0.25, 95% CI: 0.16, 0.38; *P* < 0.001) (Fig. 5).

Subgroup analysis based on the infant age showed that, tenofovir was associated with a significant lower rate of infant HBsAg positivity than other treatments when the infants were at the time of birth (RR = 0.28, 95% CI: 0.17, 0.48; *P* < 0.001), 6 months of age (RR = 0.16, 95% CI: 0.05, 0.52; *P* = 0.002), or 12 months of age (RR = 0.22, 95% CI: 0.08, 0.62; *P* = 0.004) (Fig. 5).

#### Infant HBeAg positivity

Five studies reported the data of infant HBeAg positivity [20, 36, 37, 40, 41]. The pooled estimate demonstrated that, infants in the tenofovir group had a significant lower rate of HBeAg positivity compared with the control group (RR = 0.26, 95% CI: 0.14, 0.48; *P* < 0.001).

Subgroup analysis based on the infant age suggested that, tenofovir was associated with a significant lower rate of infant HBeAg positivity than other treatments when the infants were at the time of birth (RR = 0.39, 95% CI: 0.18, 0.86; *P* = 0.020), 6 months of age (RR = 0.17, 95% CI: 0.04, 0.66; *P* = 0.010), or 12 months of age (RR = 0.18, 95% CI: 0.04, 070; *P* = 0.013).

#### Infant HBV DNA positivity

Three studies reported the data of infant HBV DNA positivity [36, 40, 41]. The pooled result showed that, the infants in the tenofovir group had a significant lower rate of HBV DNA positivity compared with the control group (RR = 0.15, 95% CI: 0.07, 0.31; *P* < 0.001).

Subgroup analysis based on the infant age indicated that, tenofovir was associated with a significant lower rate of infant HBV DNA positivity than other treatments when the infants were at the time of birth (RR = 0.39, 95% CI: 0.08, 0.39; *P* = 0.020), 6 months of age (RR = 0.13, 95% CI: 0.02, 0.88; *P* = 0.036), or 12 months of age (RR = 0.06, 95% CI: 0.00, 0.83; *P* = 0.043).

### Immunoprophylaxis failure

Three studies reported the data of immunoprophylaxis failure [37–39]. The pooled estimate demonstrated that there was a significant lower rate of immunoprophylaxis failure in infants whose mothers received tenofovir compared with these whose mothers received other treatments (RR = 0.31, 95% CI: 0.13, 0.73; *P* = 0.008).

### Maternal safety and infant safety outcomes

All the included studies reported the data of maternal and infant safety outcomes [20, 36–43]. The maternal and infant adverse events were summarized in Table 2. Pooled estimate showed that the incidences of maternal or infant adverse events were not significant difference between the tenofovir and other treatment groups.

**Table 2:** Summarized risk ratios (RR) with 95% confidence intervals (95% CIs) for adverse events

Adverse events	RR (95% CIs)	*P* value
Maternal adverse events		
Fatigue	1.03 (0.65, 16.25)	0.586
Cough	1.42 (0.60, 3.39)	0.983
Diarrhea	3.61 (0.75, 17.41)	0.424
Fever	1.56 (0.40, 6.17)	0.110
Nausea	2.06 (0.19, 22.37)	0.523
Pruritus	0.41 (0.08, 2.08)	0.552
Palpitation	0.34 (0.01, 8.33)	0.283
Dyspepsia	3.09 (0.13, 74.99)	0.511
Rash	0.95 (0.28, 3.19)	0.488
Insomnia	3.09 (0.13, 74.99)	0.928
Dizziness	0.52 (0.05, 5.59)	0.488
Abdominal pain	3.09 (0.13, 74.98)	0.586
Upper respiratory infection	0.34 (0.04, 3.25)	0.731
Gestational diabetes	3.41 (0.15, 79.47)	0.351
Arrhythmia	3.25 (0.28, 46.72)	0.612
Infant adverse events		
Hypospadias	2.78 (0.12, 67.39)	0.529
Torticollis	1.85 (0.17, 20.08)	0.612
Umbilical hernia	2.78 (0.12, 67.39)	0.529
Pneumonia	4.64 (0.23, 95.24)	0.470
Bronchitis	0.31 (0.01, 7.49)	0.948
Birth weight < 2500 g	1.10 (0.07, 16.43)	0.185
Fetal growth retardation	0.79 (0.15, 21.34)	0.546
Congenital malformation	2.34 (0.25, 64.7)	0.445

### Publication bias

We used the Begg and Egger’s test to evaluate the publication bias. The results showed that there was no evidence of significant publication bias (Egg test, *P* = 0.625; Begg test, *P* = 0.458).

## Discussion

This study is a meta-analysis with the objective of assessing the efficacy of tenofovir in reducing mother-to-infant HBV transmission, as well as monitoring safety for mothers and infants. Our study suggested that tenofovir was associated with significant reductions in maternal HBV DNA levels, infant HBsAg/HBeAg positivity, infant HBV DNA positivity, and immunoprophylaxis failure. Moreover, tenofovir induced a similar incidence of ALT, CK, and Cr elevation with other treatments. This study indicated that tenofovir was effective and safe for both mother and infant in the prevention of vertical transmission of HBV.

In this meta-analysis, we found that tenofovir significantly reduced the maternal HBV DNA levels. This finding was in consistent with the results of other studies available about the efficacy of tenofovir in preventing vertical transmission [20, 36, 40, 41]. In a prospective, multicenter trial conducted in China [36], the authors enrolled 118 pregnant patients who had chronic HBV and HBV DNA levels ≥7.5 log_10_ IU/ mL. These patients received tenofovir 300 mg daily (*N* = 62, HBV DNA 8.18 ± 0.47 log_10_ IU/mL), or no medication (*N* = 56, HBV DNA 8.22 ± 0.39 log_10_ IU/mL) [36]. At the time of delivery, the mean reduction of HBV DNA in the two groups were 3.89 ± 0.87 and 0.11 ± 0.51 log_10_ IU/mL, respectively [36], which indicated that tenofovir had a greater reduction in maternal HBV DNA than no medication. Similarly, in another multi-center, prospective opt-in observational study from Austria [20], the authors also reported greater decrease of HBV DNA in the tenofovir group. In that study, pregnant women with high viral load (>7 log IU/mL) were treated with tenofovir (*N* = 58), lamivudine (*N* = 52), or no therapy (*N* = 20). The mean reduction of HBV DNA in tenofovir group was 3.64 ± 0.9 log IU/mL, compared with 2.81 ± 1.33 in the lamivudine group [20]. This result indicated that tenofovir had a significant greater reduction of HBV DNA levels than lamivudine.

However, the advantage effect of tenofovir over other treatments in reducing HBV DNA levels was not observed in a prospective cohort study conducted in China [40]. In that study, 153 chronic HBV infectious mothers were assigned into the tenofovir group (*N* = 30) and telbivudine group (*N* = 79) [40]. At the time of delivery, the reductions of HBV DNA levels in these groups were 4.64 ± 0.5 and 3.54 ± 0.8 log IU/mL, respectively. Although tenofovir seemed to have a greater reduction in HBV DNA levels than telbivudine, the difference was not significant. Owning to the limited available data, we did not perform subgroup analysis to explore whether tenofovir had superior effect than telbivudine in reducing the HBV DNA levels.

In this meta-analysis, our results suggested that tenofovir significantly reduced the mother-to-infant transmission compared with other treatments. The use of tenofovir significantly reduced the risk of infant HBsAg positivity by 75% (RR = 0.25, 95% CI: 0.16, 0.38), the infant HBeAg positivity by 74% (RR = 0.26, 95% CI: 0.14, 0.48), and the infant HBV DNA positivity by 85% (RR = 0.15, 95% CI: 0.07, 0.31). Our results were in consistent with the study conducted by Chen WJ, et al. [40]. In that study, 3.3 and 10.0% of infants in tenofovir group had HBsAg and HBeAg positivity, as compared with 40.9 and 43.2% of infants in the control group, respectively. Similarly, other studies also reported the reduced rates of HBsAg/HBeAg positivity by tenofovir. In one prospective trial in China, the rates of HBsAg and HBV DNA positivity in tenofovir group were 1.5 and 6.15%, as compared with 10.7 and 31.48% in the control group, respectively [36]. In another multicenter, open-label, randomized, parallel-group trial in China [37], a striking decline in HBsAg positivity rate was seen from the tenofovir treatment. According to that study, 5.2% (5/97) of infants in the tenofovir group had HBsAg positivity, as compared with 18% (18/100) of infants in the control group (*P* = 0.007). These results indicated that tenofovir was effective in reducing the risk of HBV transmission from their mothers.

Regarding the immunoprophylaxis failure, our study showed that infant in the tenofovir group had a lower immunoprophylaxis failure rate than that in the control group. This result was also observed among the included studies. In a multicenter, open-label RCT, the rate of immunoprophylaxis failure in tenofovir group was 5.2%, as compared with 18% in the control group, which indicated that tenofovir therapy could reduce the immunoprophylaxis failure rate [37]. In another two cohort studies [20, 39] that evaluated the efficacy and safety of tenofovir use in pregnant HBV women, none of the infants of the tenofovir-treated mothers had immunoprophylaxis failure; whereas, 2 of 23 (8.7%) and 1 of 73 (1.4%) of infants in the control group had immunoprophylaxis failure [20, 39].

When the potential benefit of tenofovir is evaluated, the adverse effects of that also should be taken into consideration. These adverse effects include maternal ALT, CK, and Cr flares, and infant congenital malformation. Our results suggested that these adverse events were comparable between the tenofovir and control groups. There were several studies [18, 44] reporting data on ALT flares during and after the antiviral treatment in pregnant women; however, their results remained inconsistent. In the study that used telbivudine or lamivudine for the treatment of late pregnancy women [18], it was reported that 17.1% of the mothers in the treated group had severe hepatitis flare (ALT> 10 times the ULN) compared with 6.3% in the untreated mothers [18]. In another study that used lamivudine or tenofovir, the incidences of postpartum ALT flare (> 95 U/L) in the treatment and no-treatment groups were 40–50 and 29%, respectively [44]. The highly variable and conflicting results could be explained by the different criteria for the definition of ALT flares, different follow-up protocols, different antiviral agents, and lack of controls [44].

This meta-analysis has several potential limitations that should be considered. First, substantial heterogeneity was identified among the included studies. However, it should not be surprising given the differences in maternal age at delivery, gestational age, gravidity, HBV DNA levels, and baseline ALT. These factors may account for the heterogeneity and have an impact on the treatment effect. Second, some of the included studies have a relatively small sample size (*N* < 50). Compared with larger trials, studies with small sample size are more likely to overestimate the treatment effect. Third, among the nine included studies, only one was RCT, and the remaining seven were cohort studies. Despite the cohort studies can reflect the “real-world” and further support the conclusion, the cohort data may be inclined to bias because of the patient selection. Fourth, most of the included studies were conducted in China. Thus, these findings were not representative of experts’ prescribing practices all around world. Therefore, physicians should interpret our results with caution when applying them into the clinical practice.

## Conclusion

In conclusion, the current meta-analysis suggested that, tenofovir was effective in preventing the vertical transmission of HBV, as well as reducing the HBV DNA levels in HBV-infected mothers. Moreover, tenofovir was safe and tolerable for both mothers and their infants. However, considering the potential limitations in this study, more large-scale, well-designed RCTs are needed to verify these findings.

## Abbreviations

CI: Confidence interval
HBeAg: Maternal hepatitis B e antigen
HBsAg: Hepatitis B surface antigen
HBV: Chronic hepatitis B virus
RCT: Randomized controlled trial
RR: Risk ratio
WMD: Weight mean difference
